# The relationship between gastric cancer, its precancerous lesions and bile reflux: A retrospective study

**DOI:** 10.1111/1751-2980.12858

**Published:** 2020-04-21

**Authors:** Dan Li, Jian Zhang, Wen Zhu Yao, Dong Lin Zhang, Chen Chen Feng, Qi He, Huan Huan Lv, Ya Ping Cao, Jie Wang, Ying Qi, Si Ran Wu, Na Wang, Jing Zhao, Yong Quan Shi

**Affiliations:** ^1^ Xi'an Medical University Xi'an Shaanxi Province China; ^2^ State Key Laboratory of Cancer Biology, National Clinical Research Center for Digestive Diseases, Xijing Hospital of Digestive Diseases Air Force Military Medical University Xi'an Shaanxi Province China; ^3^ Division of General Medicine The First Affiliated Hospital of Xi'an Medical University Xi'an Shaanxi Province China; ^4^ Division of Gastroenterology Second Affiliated Hospital of Xi'an Jiaotong University Xi'an Shaanxi Province China

**Keywords:** bile reflux, gastric neoplasms, gastritis, intestinal metaplasia, precancerous lesions

## Abstract

**Objective:**

To evaluate the relationship between gastric cancer (GC) and precancerous lesions and bile reflux.

**Methods:**

Medical records of 30 465 participants who underwent gastroscopy between January and December 2018 in our center were reviewed. Their age, sex, time of endoscopy, endoscopic/histologic diagnosis and grade of bile reflux were recorded. The participants were further divided into the chronic gastritis group (n = 27 807), a precancerous lesion group (n = 1943) and a GC group (n = 715). The χ^2^ tests and hierarchical analyses were performed.

**Results:**

Patients aged 18‐27 years had a higher bile reflux rate than those aged 28‐37 and 68‐75 years (*P* < 0.001), while it did not differ between patients aged <50 years and those over 50 years (*P* = 0.639). It was lower in men than in women (*P* < 0.001). The bile reflux rate did not differ in terms of months, seasons and half of the year (all *P* > 0.05), but differed between morning and afternoon when they underwent the endoscopy (*P* = 0.000). There was an interrelationship between the severity of gastric mucosal disease and bile reflux grade (*r* = 0.171). After excluding the effects of sex, age and time of endoscopy on bile reflux, bile reflux rate in chronic gastritis and precancerous lesions was lower than in gastric cancer (*P* < 0.01).

**Conclusions:**

Bile reflux may be a risk factor for gastric cancer and precancerous lesions. A high grade of bile reflux may be associated with the progression of gastric mucosal diseases.

## 1 INTRODUCTION

As the fifth most common malignant tumor worldwide, more than one million new cases of gastric cancer (GC) were estimated to be diagnosed in 2018; while the cancer‐related mortality rate of GC is the third highest (equivalent to one in every 12 deaths worldwide).^1^ In China the incidence and mortality of GC are also high.^2^ According to the Correa model, despite its complex etiology GC usually begins with atrophic gastritis, intestinal metaplasia or atypical hyperplasia, and progresses to carcinogenesis.^3^ *Helicobacter pylori* (*H. pylori*) infection, patient’s age, sex, race, genetic background, dietary habits, and psychological factors are risk factors related to the occurrence and development of GC.^4^

Nearly 90% of patients with non‐cardiac GC are infected with *H. pylori*.^5^ Systematic reviews and meta‐analyses have demonstrated that the eradication of *H. pylori* can effectively reduce the incidence of GC by 34%‐54%.^6^, ^7^ Other studies have found that eradication of *H. pylori* can partially reverse gastric atrophy but cannot reverse intestinal metaplasia.^8^, ^9^ Therefore, the eradication of *H. pylori* cannot completely prevent the occurrence of GC. We believe that there are other important factors that affect the occurrence of GC, apart from *H. pylori* infection.

Duodenogastric reflux, also known as bile reflux, refers to the backflow of duodenal contents, including bile, pancreatic juice and duodenal juice, into the stomach. It is a common pathophysiological phenomenon in the digestive tract. Bile is thought to be the main duodenal component. Bile reflux has been found to be closely related to upper gastrointestinal inflammation, development of ulcers, intestinal metaplasia and carcinoma.^10^, ^11^, ^12^ A previous study of 2283 patients found that high levels of bile acid reflux were linked to an increased risk of intestinal metaplasia.^12^ Thus, we hypothesized that bile reflux might play an important role in the occurrence and development of GC and precancerous lesions. Therefore, we conducted this study to evaluate the association between bile reflux and GC and the precancerous lesions.

## 2 PARTICIPANTS AND METHODS

### 2.1 Participants

We retrospectively reviewed the medical records of 36 179 individuals who underwent a gastroscopy without anesthesia between January 2018 and December 2018 at our hospital. We included adult patients aged between 18 and 75 years who were diagnosed as endoscopically or histologically confirmed chronic gastritis and/or intestinal metaplasia, mild to moderate atypical hyperplasia, or those having a definite diagnosis of GC. The exclusion criteria were: (a) with benign gastric or duodenal ulcers (n = 2042); (b) had undergone upper gastrointestinal surgery (n = 1381); (c) with esophageal cancer (n = 459), gastric mucosa‐associated lymphoid tissue (MALT) lymphoma or stromal tumors (n = 42), or duodenal cancer (n = 28); (d) acute upper gastrointestinal injury (n = 152); (e) gastric retention (n = 236); or (f) unclear identification of a mucous lake or no mucus under gastroscopy (n = 1374). Finally, a total of 30 465 patients were included in the analysis. The research protocol was approved by the Institutional Ethics Committee of our hospital, and was conducted in accordance with the Declaration of Helsinki. Written informed consent from the included patients was waived due to the retrospective nature of this study.

### 2.2 Diagnosis and patient grouping

All patients underwent white‐light endoscopy (WLE); for those suspected of having abnormal findings under WLE, narrow band imaging was performed to further identify the lesions. For patients highly suspected of having GC under endoscopy, the site of biopsy and the number of biopsy specimens were decided by an endoscopic physician according to the location and size of the lesion. Five biopsy specimens (two from the gastric corpus, one from the gastric angle and two from the gastric antrum) were obtained from non‐GC patients with obvious intestinal metaplasia under gastroscopy based on the new Sydney system for the classification of chronic gastritis. For non‐GC patients who had obvious lesions other than intestinal metaplasia under endoscopy, such as localized erosion, patches of changed color and superficial protrusions, one to five biopsy specimens were taken as determined by the endoscopic physician; while no biopsy was performed in when no obvious lesions under endoscopy.

The enrolled participants were further divided into three groups: (a) the chronic gastritis group (n = 27 807), including patients diagnosed as chronic atrophic gastritis or chronic non‐atrophic gastritis, without precancerous lesions under both gastroscopy and histological examination; (b) the precancerous lesion group (n = 1943), including patients with intestinal metaplasia, or mild to moderate atypical hyperplasia, after those with histologically diagnosed severe atypical hyperplasia were excluded; and (c) the GC group (n = 715).^13^, ^14^

**FIGURE 1:**
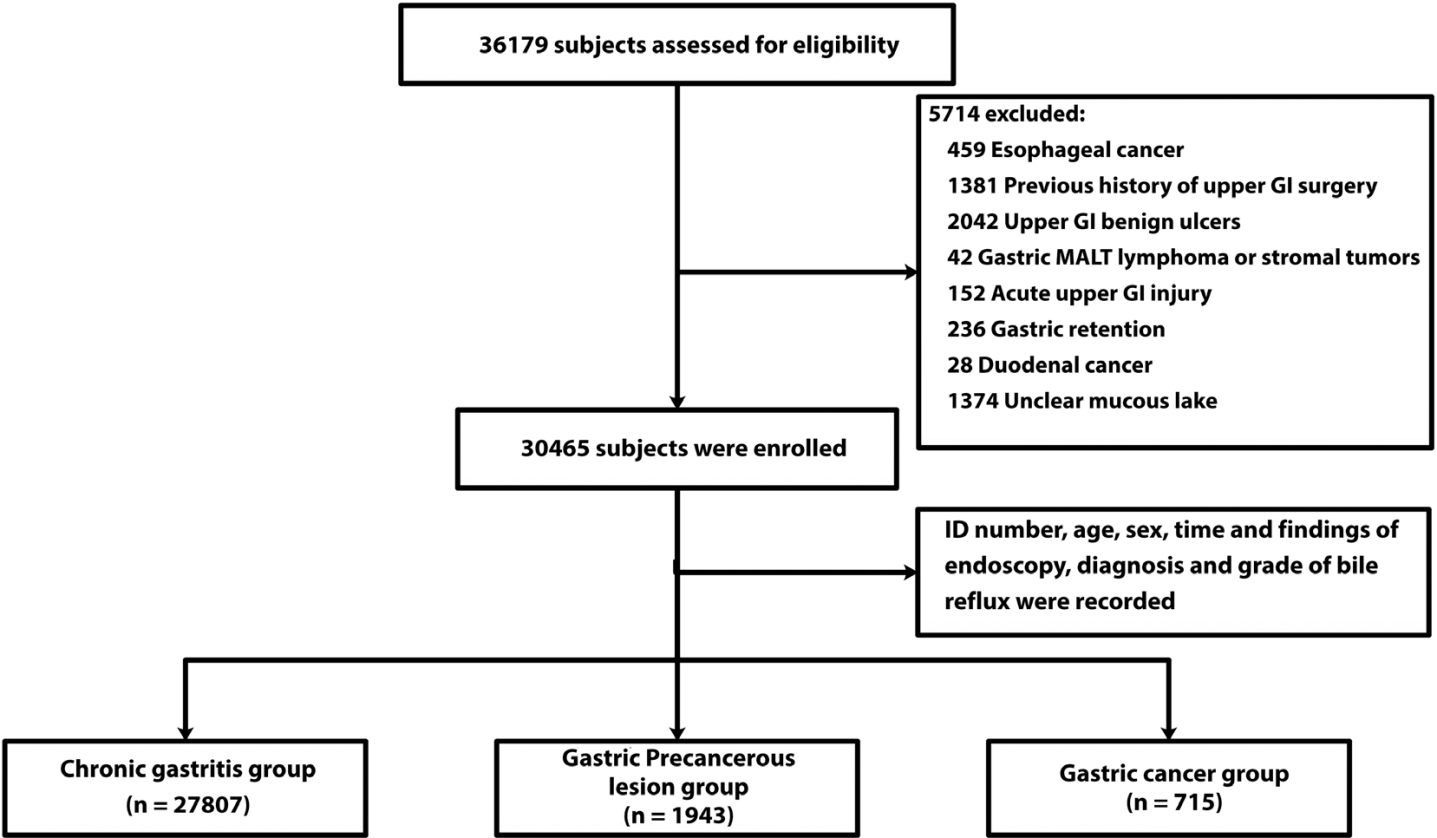
Flowchart of participant enrollment

The ID number, age and sex of the patients, time and findings of the endoscopy, their diagnosis and grade of bile reflux were recorded. The flow diagram of the study design is shown in Figure 1.

### 2.3 Endoscopic findings and the grade of bile reflux

**FIGURE 2:**
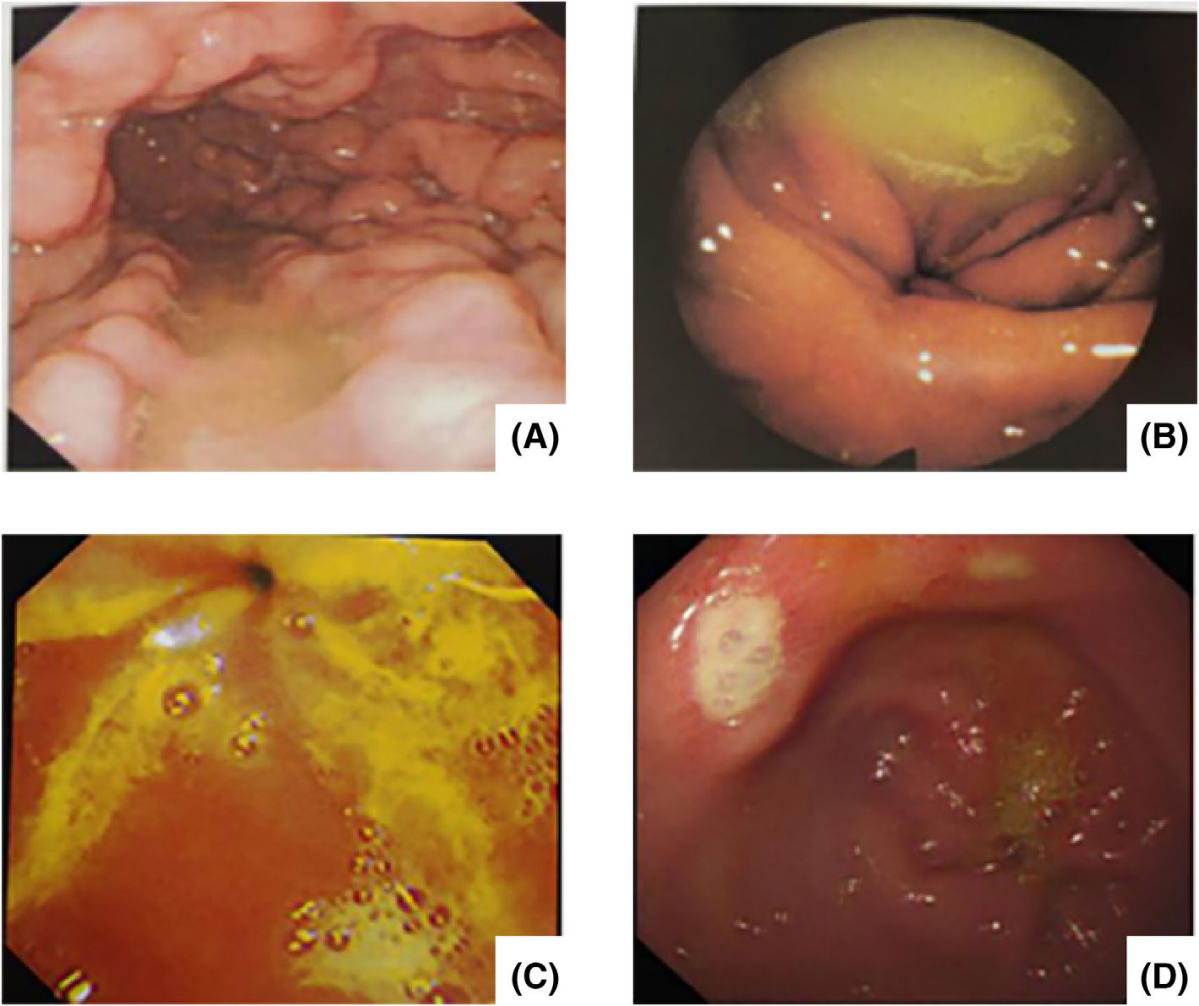
Grades of bile reflux. A, grade I bile reflux; B, grade II bile reflux; C, grade III bile reflux; and D, transient bile reflux [Color figure can be viewed at wileyonlinelibrary.com]

All the endoscopic images were reviewed by two endoscopic physicians. The grade of bile reflux was classified based on the status of the mucous lake under gastroscopy as follows: no bile reflux, clear mucous lake; grade I bile reflux, light yellow mucous lake; grade II bile reflux, yellowish green mucous lake; and grade III bile reflux, dark yellow, viscous and turbid mucous lake with a bile spot block.^15^ Some patients who were diagnosed as transient bile reflux, namely, fresh bile reflux that manifests as reflux bile floating on the gastric mucous lake, were also treated though they were classified as bile‐free (Figure 2).

### 2.4 Statistical analysis

Statistical analyses were performed by using the SPSS software version 23.0 (IBM, Armonk, NY, USA). Continuous variables were expressed as mean ± standard deviation, whereas categorical variables were expressed as numbers and percentages or frequencies. The χ^2^ test was used to compare the rate of bile reflux between the groups, while one‐way ANOVA was used for the comparison of continuous variables. The Spearman's correlation coefficient was used, and the relationship between GC or precancerous lesions and grade of bile reflux was evaluated. *P* value <0.05 was considered statistically significant.

## 3 RESULTS

### 3.1 Bile reflux and its association with age, sex, season and time of fasting

**FIGURE 3:**
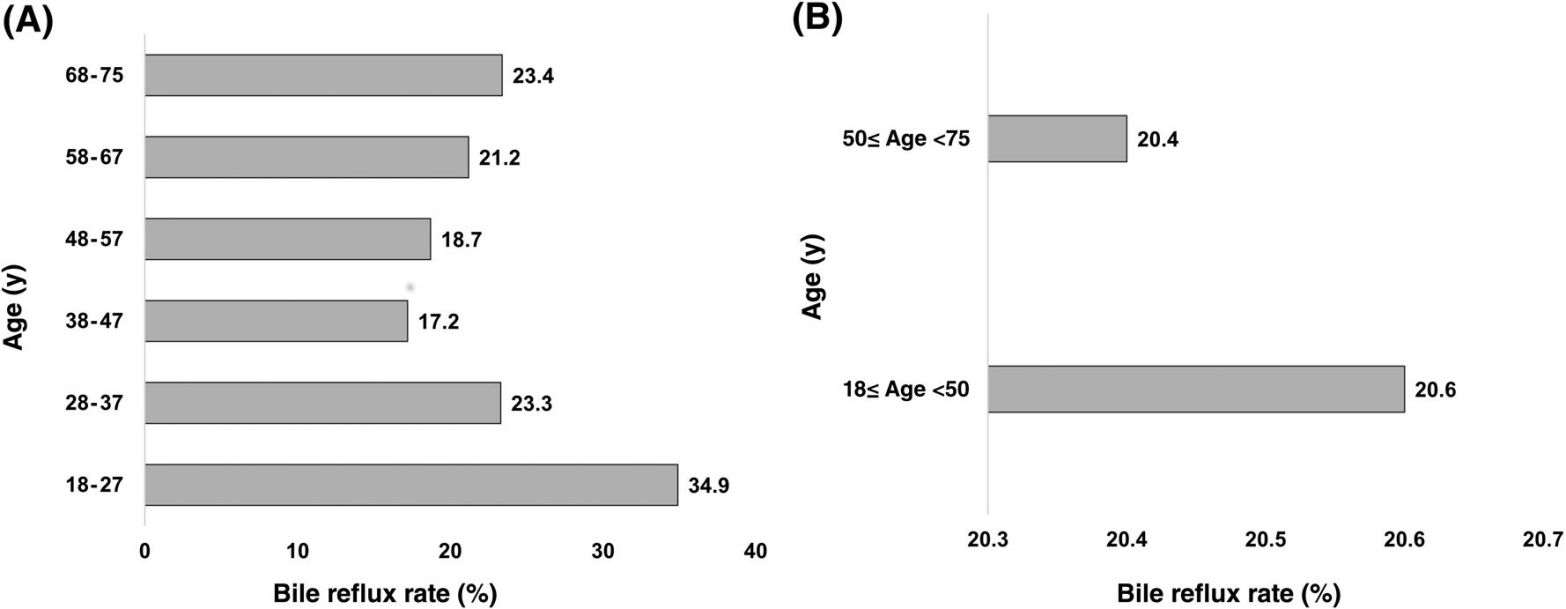
Relationship between bile reflux and age by comparing the rate of bile reflux among A, six different age groups and B, 18‐<50 years vs 50‐<75 years

Among all the 30 465 patients, 6235 were diagnosed as having bile reflux, resulting in an overall rate of 20.5%. As shown in Figure 3, the overall rate of bile reflux was the highest (34.9%) among patients aged 18‐27 years, followed by those aged 68‐75 years (23.4%), and those aged 28‐37 years (23.3%), respectively. The lowest rate of bile reflux was observed in patients between 38 and 47 years old (17.2%). There was statistically significant differences among the six age groups (*P* < 0.001). However, the rate of bile reflux did not significantly differ between patients aged 18‐<50 years and those aged 50‐<75 years (20.6% vs 20.4%, *P* = 0.639).

We analyzed the impact of sex on the rate of bile reflux, and found that bile reflux was detected in 3099 (19.7%) of 15 742 male patients and 3136 (21.3%) of 14 723 female patients. The rate in men was significantly lower than that in women (*P* < 0.001).

We then divided the time when the gastroscopy was performed (eg, the time of fasting) into morning (8:00‐11:30) and afternoon (13:00‐16:00). A total of 17 761 patients underwent a gastroscopy in the morning, of whom 3185 had bile reflux, with a rate of 17.9%. Of the 12 704 patients who underwent a gastroscopy in the afternoon, 3050 had bile reflux, and the rate of bile reflux was calculated to be 24.0%. There was a noticeable difference in bile reflux rates between the morning and afternoon groups (*P* < 0.001).

**FIGURE 4:**
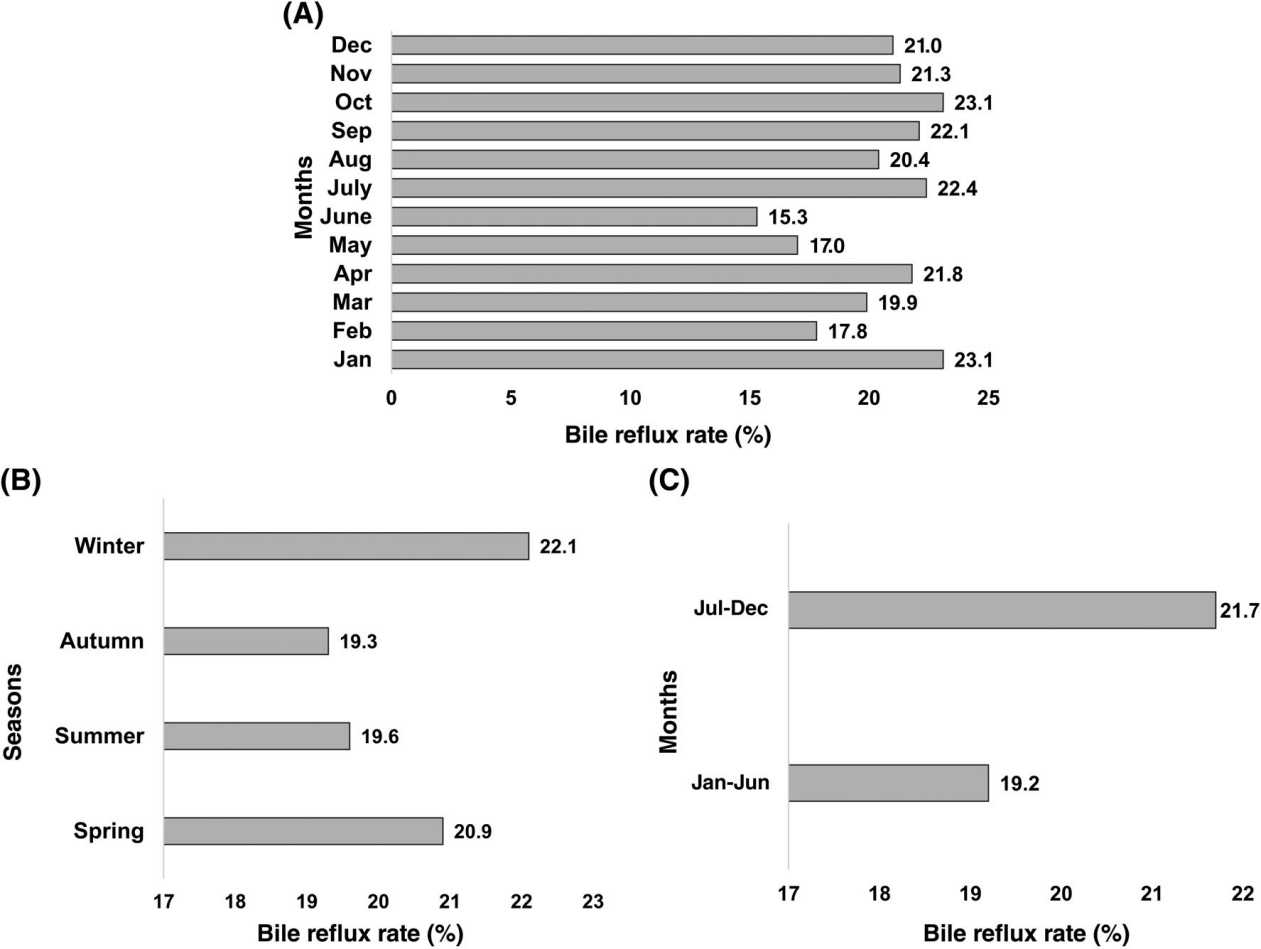
Rates of bile reflux at A, different months; B, spring to winter; and C, Jan‐June and July‐Dec

As shown in Figure 4, there was no noticeable difference in the rate of bile reflux at different months (*P* = 0.240) and seasons (*P* = 0.213), or between January to June and July to December (*P* = 0.157).

### 3.2 Impact of bile reflux on gastric mucosal lesions

#### 3.2.1 *Association between bile reflux and gastric mucosal lesions*

As indicated in Table 1, the rate of bile reflux in the chronic gastritis group (18.4% [5118/27807] vs 57.3% [410/715]) and precancerous lesion group (36.4% [707/1943] vs 57.3% [410/715]) was significantly lower than that in GC group (both *P* < 0.001). Bile reflux in the chronic gastritis group was significantly less common than that in the group with precancerous lesions as well (*P* < 0.001). The Spearman’s correlation analysis was then used to determine whether there was a linear relationship between gastric lesions and the grade of bile reflux. As shown in Table 2, the Spearman’s correlation coefficient of the grade of bile reflux and the three groups was 0.171 (*P* < 0.01), indicating that the grade of bile reflux was positively correlated with the severity of gastric lesions.

**TABLE 1:** Patients’ characteristics

	Chronic gastritis	Precancerous lesion	Gastric cancer	*P* value
	(N = 27 807)	(N = 1943)	(N = 715)	
Age, y (mean ± SD)	48.34 ± 11.90	54.52 ± 9.87	57.31 ± 10.68	<0.001
Sex, n (%)				<0.001
Male	14 026 (50.4)	1196 (61.6)	520 (72.7)	
Female	13 781 (49.6)	747 (38.4)	195 (27.3)	
Bile reflux, n (%)				<0.001
Yes	5118 (18.4)	707 (36.4)	410 (57.3)	
No	22 689 (81.6)	1236 (63.6)	305 (42.7)	

Abbreviation: SD, standard deviation.

**TABLE 2:** Association between the grade of bile reflux and gastric mucosal lesions (n, %)

	No bile reflux	Grade of bile reflux				*P* value
		I	II	III	*r*	
Chronic gastritis (N = 27 807)	22 689 (81.60)	2685 (9.66)	1753 (6.30)	680 (2.44)	0.171	<0.01
Precancerous lesion (N = 1943)	1236 (63.61)	365 (18.78)	202 (10.40)	140 (7.21)		
Gastric cancer (N = 715)	305 (42.66)	177 (24.76)	126 (17.62)	107 (14.96)		

Grade I, light yellow mucous lake; grade II, yellowish green mucous lake; grade III, dark yellow, viscous and turbid mucous lake with a bile spot block.

#### 3.2.2 *Exclusion of the effects of sex, age, and time of fasting on bile reflux*

As shown in Table 1, there were noticeable differences in sex and age among the three groups. Therefore, we further investigated the rates of bile reflux when stratified according to the patients' age and sex. Regardless of patients’ age (Table 3) and sex (Table 4), the rate of bile reflux in chronic gastritis and precancerous lesions was noticeably lower than that in GC. Moreover, the bile reflux rate of the precancerous lesion group was significantly higher than that in the chronic gastritis group.

**TABLE 3:** Rates of bile reflux among patients in different age groups (n, %)

	18‐<50 y		50‐<75 y	
	Bile reflux	No bile reflux	Bile reflux	No bile reflux
Chronic gastritis	2762 (19.5)	11 431 (80.5)	2356 (17.3)	11 258 (82.7)
Precancerous lesion	220 (38.6)*	350 (61.4)	487 (35.5)*	886 (64.5)
Gastric cancer	96 (58.9)* ^,^ **	67 (41.1)	314 (56.9)* ^,^ **	238 (43.1)

* *P* < 0.01 vs chronic gastritis.

** *P* < 0.01 vs precancerous lesion.

**TABLE 4:** Rate of bile reflux by sex (n, %)

	Men		Women	
	Bile reflux	No bile reflux	Bile reflux	No bile reflux
Chronic gastritis	2369 (16.9)	11 657 (83.1)	2749 (19.9)	11 032 (80.1)
Precancerous lesion	439 (36.7)*	757 (63.3)	268 (35.9)*	479 (64.1)
Gastric cancer	291 (56.0)* ^,^ **	229 (44.0)	119 (61.0)* ^,^ **	76 (39.0)

* *P* < 0.01 vs chronic gastritis.

** *P* < 0.01 vs precancerous lesion.

Because bile reflux is known to be related to the time of fasting, we performed a stratified analysis based on fasting in the morning and in the afternoon. As shown in Table 5, regardless of the time when the gastroscopy was performed, the rate of bile reflux in the three groups was noticeably different (*P* < 0.01). The GC group had the highest bile reflux rate (morning: 57.0% and afternoon: 57.7%), followed by the precancerous lesion group (morning: 34.9% and afternoon: 38.3%). The chronic gastritis group had the lowest bile reflux rate (morning: 15.9% and afternoon: 22.0%). The rates of bile reflux in the chronic gastritis and precancerous lesion groups were significantly lower than those in the GC group (*P* < 0.01 and *P* < 0.001, respectively). While the bile reflux rate of the precancerous lesion group was higher than that in the chronic gastritis group and the difference was statistically significant (*P* < 0.01). These results suggested that bile reflux may be a risk factor for gastric mucosal lesions and was independent of age, sex, and time of fasting.

**TABLE 5:** Rate of bile reflux in different groups by time of fasting (n, %)

	8:00‐11:30 am		13:00‐16:00 pm	
	Bile reflux	No bile reflux	Bile reflux	No bile reflux
Chronic gastritis	2579 (15.9)	13 690 (84.1)	2539 (22.0)	8999 (78.0)
Precancerous lesion	387 (34.9)*	721 (65.1)	320 (38.3)*	515 (61.7)
Gastric cancer	219 (57.0)* ^,^ **	165 (43.0)	191 (57.7)* ^,^ **	140 (42.3)

* *P* < 0.01 vs chronic gastritis.

** *P* < 0.01 vs precancerous lesion.

## 4 DISCUSSION

Many studies have explored whether bile reflux is associated with gastric diseases. In 1968 bile acid was reported to destroy gastric mucosa.^16^ The degree of gastric mucosal damage caused by bile acid increases as the pH of gastric juice decreases.^17^ In addition, some studies have revealed that bile reflux plays a role in the development of residual GC after a partial gastrectomy,^18^, ^19^ especially among patients who have undergone the Billroth II procedure.^20^ It has also been reported that bile reflux after a Billroth II gastrectomy may be an important risk factor for residual GC.^21^ However, the relationship between bile reflux and GC still needs further investigation.

Bile reflux is usually divided into primary and secondary reflux. Bile reflux in patients without a previous history of gastroduodenal surgery is defined as primary bile reflux, while that after gastric surgery is defined as secondary bile reflux. All patients in our study had primary bile reflux. Our results indicate that bile reflux is related to patients’ age, sex and fasting time. The rate of bile reflux in men was significantly lower than that in women. Similarly, this rate was lower in middle‐aged patients than in young and elderly patients. Additionally, the bile reflux rate was lower in patients underwent endoscopy in the morning than in those having endoscopy in the afternoon. There was no significant association between the month when performing gastroscopy and bile reflux. The bile reflux rate gradually increased from the chronic gastritis group to the group with precancerous lesions, and to the GC group. After stratifying the factors of sex, age and fasting time, similar results were obtained. Moreover, we found that a higher grade of bile reflux is associated with more severe damage to gastric mucosa. However, the rate of bile reflux in our study was highest among young women, but GC was more likely to occur in older men. This may be related to other risk factors such as genetic background. Our results suggest that bile reflux may be a risk factor for GC. Therefore, it is critical to provide preventive measures for those with a high risk of bile reflux.

The relationship between bile reflux and *H. pylori* infection remains unclear. One study found that bile acid that remains in gastric juice can promote ulcer healing and inhibit the growth of *H. pylori*.^22^ Another study on the etiology of cardiac cancer found that in environments in which production of acid is low, high concentrations of soluble bile acids may play the role of bactericides for *H. pylori* in the antrum.^23^ However, a study on the inflammation of the remnant stomach after a gastrectomy found that bile reflux was not related to the severity of chronic and active inflammatory cellular infiltration or *H. pylori* infection.^24^ Bile acid may affect the extent of gastric atrophy, regardless of *H. pylori* infection.^12^ A case‐control study indicated that *H. pylori* infection was detected in 35.8% of the patients with bile reflux gastritis and in 73.0% of the GC group.^25^ There have been no studies reporting the association between a family history of GC and bile reflux. Therefore, we conclude that *H. pylori* infection, family history and bile reflux might be independent of each other and speculate that all three are independent risk factors of GC. Therefore, we consider that the lack of information on *H. pylori* infection and family history in our research does not affect our results.

Intestinal metaplasia is most prevalent among patients with *H. pylori* infection and a high concentration of bile acid in gastric juice. A positive relationship has been found between bile reflux and the severity of glandular atrophy and chronic inflammation, the severity of lamina propria inflammation and foveolar hyperplasia.^26^ In another study, a high bile reflux index emerged as a significant independent factor associated with intestinal metaplasia in the cardia.^27^ Matsuhisa and Tsukui failed to find the relationship between refluxed bile acid and atrophy or intestinal metaplasia in *H. pylori*‐positive group; in contrast, intestinal metaplasia was more common in the high‐concentration bile reflux group with negative *H. pylori* infection.^28^ High bile acid concentrations in gastric juice were found to be related to an increased risk of intestinal metaplasia.^12^ Wang et al reported that chronic bile reflux can cause significant glandular gastric hyperplasia and gastric glandular dilatation and promote the formation of GC, and that intestinal metaplasia and ulcers in the glandular stomach are rare.^29^ However, many clinical trials include small sample sizes and are only limited to chronic gastritis or intestinal metaplasia. In particular, there have been no comprehensive studies reporting the relationship between bile reflux and gastritis, precancerous lesions and GC. While our study included a larger sample, which may provide a higher level of clinical evidence.

There were some limitations to our study. First, because of its retrospective study design the patients’ data were not comprehensive, information such as their *H. pylori* infection, living habits and genetic background were missing. Second, some patients might have had precancerous lesions but were wrongly included in the chronic gastritis group because they did not receive biopsy. However, this might not have effects on our results significantly as the rate of bile reflux was still higher in those with precancerous lesions than in the chronic gastritis group. Third, this was a single‐center study; therefore, our results have limited generalizability and the influence of other risk factors except bile reflux on GC cannot be ruled out. Therefore, large cross‐sectional studies are needed to determine whether bile reflux is an independent risk factor for GC and what role it may play in the progression of precancerous lesions and GC.

In conclusion, bile reflux is closely associated with the development of GC and its precancerous lesions, and may be an independent risk factor for GC. A higher rate of bile reflux may be related to a higher risk of GC and precancerous lesions. Physicians should pay more attention to patients with bile reflux in order to prevent the occurrence of GC and precancerous lesions.
